# Monitoring the Remodeling of Biohybrid Tissue‐Engineered Vascular Grafts by Multimodal Molecular Imaging

**DOI:** 10.1002/advs.202105783

**Published:** 2022-02-04

**Authors:** Elena Rama, Saurav Ranjan Mohapatra, Christoph Melcher, Teresa Nolte, Seyed Mohammadali Dadfar, Ramona Brueck, Vertika Pathak, Anne Rix, Thomas Gries, Volkmar Schulz, Twan Lammers, Christian Apel, Stefan Jockenhoevel, Fabian Kiessling

**Affiliations:** ^1^ Institute for Experimental Molecular Imaging University Clinic and Helmholtz Institute for Biomedical Engineering RWTH – Aachen University Forckenbeckstrasse 55 52074 Aachen Germany; ^2^ Department of Biohybrid & Medical Textiles Institute of Applied Medical Engineering RWTH – Aachen University Forckenbeckstrasse 55 52074 Aachen Germany; ^3^ Institute for Textile Technology RWTH – Aachen University Forckenbeckstrasse 55 52074 Aachen Germany

**Keywords:** *α*_v_ *β*_3_ integrins, molecular imaging, poly(lactic‐*co*‐glycolic acid), superparamagnetic iron‐oxide nanoparticles, tissue‐engineering

## Abstract

Tissue‐engineered vascular grafts (TEVGs) with the ability to grow and remodel open new perspectives for cardiovascular surgery. Equipping TEVGs with synthetic polymers and biological components provides a good compromise between high structural stability and biological adaptability. However, imaging approaches to control grafts’ structural integrity, physiological function, and remodeling during the entire transition between late in vitro maturation and early in vivo engraftment are mandatory for clinical implementation. Thus, a comprehensive molecular imaging concept using magnetic resonance imaging (MRI) and ultrasound (US) to monitor textile scaffold resorption, extracellular matrix (ECM) remodeling, and endothelial integrity in TEVGs is presented here. Superparamagnetic iron‐oxide nanoparticles (SPION) incorporated in biodegradable poly(lactic‐*co*‐glycolic acid) (PLGA) fibers of the TEVGs allow to quantitatively monitor scaffold resorption via MRI both in vitro and in vivo. Additionally, ECM formation can be depicted by molecular MRI using elastin‐ and collagen‐targeted probes. Finally, molecular US of *α*_v_ *β*_3_ integrins confirms the absence of endothelial dysfunction; the latter is provocable by TNF‐*α*. In conclusion, the successful employment of noninvasive molecular imaging to longitudinally evaluate TEVGs remodeling is demonstrated. This approach may foster its translation from in vitro quality control assessment to in vivo applications to ensure proper prostheses engraftment.

## Graphical abstract

Noninvasive imaging modalities are fundamental to control the remodeling and function of biohybrid tissue‐engineered vascular grafts, which is particularly true for the critical transition time between late in vitro maturation and early in vivo engraftment. Here, a noninvasive multimodal, molecular imaging concept is presented to longitudinally monitor textile scaffold resorption, extracellular matrix remodeling, and endothelial inflammation.

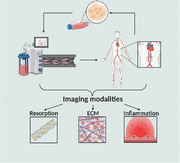

## 1 Introduction

Despite major discoveries and successes that occurred in the field of prevention and treatment of cardiovascular diseases, they remain the leading cause of death worldwide.^[^ ^1^, ^2^ ^]^ Cardiovascular pathologies are associated with a broad spectrum of comorbidities, including partial occlusion or complete loss of patency, aneurism of the vessel wall, reduced blood supply, and consequently insufficient nutrients uptake leading to tissue damage and inflammation.

Tissue engineering (TE) has the potential to improve, repair, or replace partially or completely damaged tissues and organs through the development of prostheses mimicking both functionally and biologically the native structures. In recent decades, enormous progress has been made in the study and development of cardiovascular implants, vascular prostheses, patches, stents, and shunts.^[^ ^3^ ^]^ About 600 000 surgical operations using such revascularization devices are performed annually. However, once implanted in the body, many of the synthetic prosthetic models clinically used today, i.e., expanded polytetrafluoroethylene (GORE‐TEX) and poly(ethylene terephthalate) (Dacron), show poor outcomes. Their lack of in vivo remodeling and growth proficiency in addition to inadequate hemocompatibility can result in complete occlusion of the prostheses, thrombus formation, calcific deposition, hyperplasia of the intima, and biomechanical properties mismatch.^[^ ^4^, ^5^, ^6^, ^7^ ^]^

TE is now focusing on the employment of a combination of synthetic materials, which provide mechanical strength, and biological components to contribute to the biocompatibility of the prostheses with the host organism. In this context, a refined strategy to ensure high biocompatibility and functionality of vascular graft is to provide strong structural support with synthetic material at the early post‐transplantation phase to avoid graft rupture, but also to have parts of the scaffold being degraded to maintain a mechanical trigger stimulating extracellular matrix (ECM) production and minimizing the amount of synthetic material finally remaining in the body. This can be achieved by combining a nondegradable with a biodegradable scaffold material. In this view, poly(lactic‐*co*‐glycolic acid) (PLGA) is becoming one of the most used synthetic materials for the fabrication of TE prostheses.^[^ ^8^, ^9^ ^]^ Moreover, it is a Food and Drug Administration (FDA) approved, biocompatible and biodegradable copolymer, characterized by intrinsic favorable mechanical capabilities, a tailorable biodegradation rate, and the release of nontoxic degradation products.^[^ ^10^ ^]^ However, the accumulation of these degradation products, i.e., lactic and glycolic acid, raises several concerns especially because they trigger the decrease of the pH in the surrounding environment leading to swelling of the tissues as well as inflammatory (acute and chronic) and foreign body reactions.^[^ ^11^ ^]^ Therefore, there is a high demand to intensify the investigation of prostheses’ behavior and performances upon in vivo implantation.

The implementation of noninvasive and nondestructive monitoring tools is highly desirable as they could strongly foster clinical translation of TE implants and enable the early detection of therapeutically reversible dysfunctions, i.e., damage on the integrity of the prostheses and loss of functionality. From this point of view, magnetic resonance imaging (MRI) is an attractive imaging modality because, unlike computed tomography (CT) and X‐ray, it does not require ionizing radiation and is characterized by high spatial and temporal resolution with excellent soft‐tissue contrast. Similarly, ultrasound (US) is widely used in the clinic because it provides outstanding real‐time diagnostic and imaging information with high patient safety.^[^ ^12^, ^13^, ^14^ ^]^

Nonetheless, conventional MRI and US are inadequate to monitor textile scaffold components and cellular behavior within TE implants. Novel approaches toward the incorporation of MRI contrast agents into the textile scaffold and cells embedded into the structure of vascular grafts have already been investigated and proved to be efficient for their visualization.^[^ ^4^, ^15^, ^16^ ^]^ However, comprehensive diagnostic tools for monitoring implant remodeling from late in vitro bioreactor conditioning to early in vivo implantation, being the most critical phase, are still hardly available.

**Figure 1:**
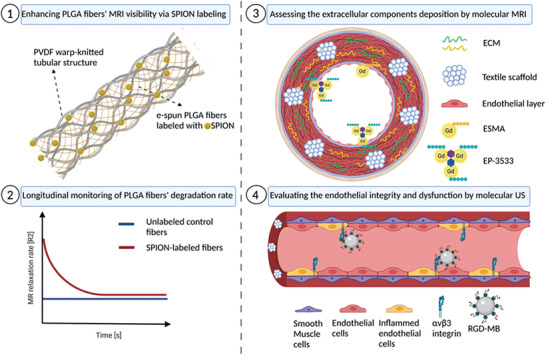
Schematic depiction of the study design (created with BioRender.com).

In this regard, this study aimed to develop imageable biohybrid vascular scaffolds and a comprehensive molecular imaging concept to monitor their late in vitro conditioning and the early in vivo implantation phase. The imaging concept included the assessment of the textile scaffold remodeling, deposition of main ECM components, and the onset of potential inflammatory reactions. As shown in **Figure** **1**, we first developed tissue‐engineered vascular grafts (TEVGs) consisting of nondegradable warp‐knitted polyvinylidene fluoride (PVDF) tubular meshes coated with degradable and biocompatible e‐spun PLGA fibers that were molded into fibrin gel containing smooth muscle cells (SMCs) and endothelial cells (ECs). Additionally, superparamagnetic iron oxide nanoparticles (SPION) were passively incorporated into the PLGA fibers allowing the longitudinal monitoring of the degradable copolymer by MRI. Furthermore, the replacement of the degraded fibers by deposition of newly synthesized ECM components was assessed via means of elastin‐ and collagen type I‐targeted MRI molecular gadolinium‐based probes (ESMA and EP‐3533). Moreover, molecular ultrasound imaging with c[RGDfK]‐functionalized poly(n‐butyl cyanoacrylate) microbubbles (c[RGDfK]‐MBs) was employed to assess αvβ3 integrin expression as a marker of inflammation and endothelial dysfunction.

## 2 Results and Discussion

### 2.1 MRI Reliably Detects SPION‐Labeled Scaffolds and Captures PLGA Degradation

Despite the high biocompatibility of PLGA, its invisibility by noninvasive imaging makes it an uncontrollable component when used as scaffold material of the vascular prosthesis. This is particularly critical during the in vitro conditioning and in vivo remodeling phase.

**Figure 2:**
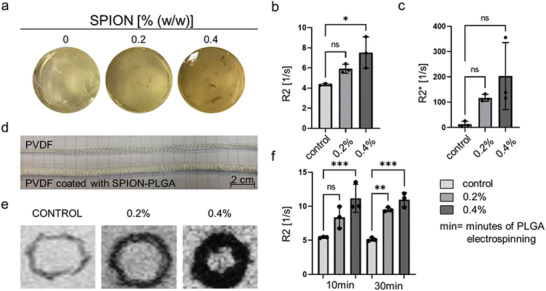
MRI investigation of SPION‐labeled PLGA fibers. a) Gelatin phantoms containing 0%, 0.2%, and 0.4% (w/w) of SPION. b,c) R2 and R2* analyses of SPION‐labeled PLGA fibers (both concentrations) in comparison to the unlabeled control. d) Visual comparison between an uncoated and SPION‐labeled PLGA‐coated PVDF tubular scaffold. e) T2 weighted MRI images of PVDF tubular scaffolds coated with 0.2% and 0.4% (w/w) of SPION. f) R2 analyses of different PLGA electrospinning time, 10 and 30 min, containing 0.2% and 0.4% (w/w) of SPION. All values were obtained in triplicates: mean ± SD; *t*‐test with Tukey post hoc correction test was applied; ns > 0.05, ^*^ *p* < 0.05, ^**^ *p* < 0.01, ^***^ *p* < 0.001, and ^****^ *p* < 0.0001.

To overcome this hurdle, we passively labeled the PLGA fibers with SPION (**Figure** **2**a). These iron‐oxide nanoparticles lead to local susceptibilities and predominantly decrease T2 and T2* relaxation times (respectively increase the R2 and R2* relaxation rates), resulting in a strong hypointense MRI contrast. The resulting R2 quantitative MRI analyses of the different PLGA samples, as depicted in Figure 2b, showed a significant improvement in fiber detectability with increasing SPION concentrations (R2 values for control: (4.3 ± 0.3) s^−1^; 0.2% SPION‐labeled PLGA fibers: (5.9 ± 0.9) s^−1^; 0.4% SPION‐labeled PLGA fibers: (7.5 ± 1.3) s^−1^). Interestingly, for R2* the difference between the samples was less prominent and not significant although, also here, a slightly increased relaxation rate was detected with higher SPION concentration (Figure 2c; R2* values for control: (12.6 ± 9.3) s^−1^; 0.2% SPION‐labeled PLGA fibers: (117 ± 10.6) s^−1^; 0.4% SPION‐labeled PLGA fibers: (203 ± 106) s^−1^). The statistical insignificance of R2* changes may be explained by the high sensitivity of the employed gradient echo sequence not only to the presence of SPION, but also to SPION unrelated inhomogeneities in the main magnetic B0 field that might have persisted even after performing field map‐based shimming. Hence, R2 was selected for all subsequent analyses.

Encouraged by the improved visibility of the SPION‐labeled PLGA fibers via MRI, the PLGA fibers, electrospun for 10 and 30 min, were subsequently evaluated in combination with the warp‐knitted PVDF tubular structures that are the main supporting and nonbiodegradable component of the textile scaffolds in the vascular graft (Figure 2d‐e). As displayed in Figure 2f, the SPION‐labeled PLGA fibers showed higher R2 relaxation rates in comparison with the unlabeled control (R2 values for 10 min of PLGA electrospinning: control: (5.4 ± 0.1) s−1; 0.2% SPION‐labeled PLGA fibers: (8.2 ± 1.4) s−1; 0.4% SPION‐labeled PLGA fibers: (11.2 ± 2.1) s−1; R2 values for 30 min of PLGA electrospinning: control: (5.1 ± 0.3) s−1; 0.2% SPION‐labeled PLGA fibers: (9.5 ± 0.3) s−1; 0.4% SPION‐labeled PLGA fibers: (10.9 ± 1.1) s−1). However, no difference was observed between the 10 and 30 min of PLGA electrospinning conditions. Therefore, the increase of the electrospinning time and hence the thickness of the PLGA coating layer do not affect the MRI detection of these fibers.

**Figure 3:**
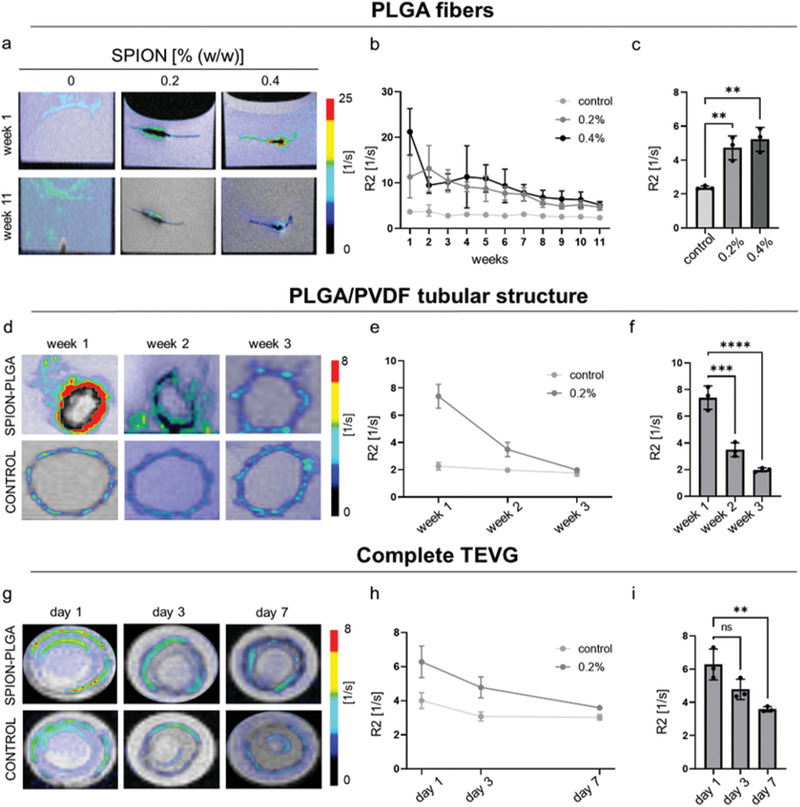
Monitoring of PLGA fiber degradation by MRI. a) Superimposed R2 color maps on T2 weighted images of 0.2% and 0.4% (w/w) SPION‐labeled PLGA fibers embedded in fibrin gel during the first week (top) and the last week (bottom) of the experiment. b) R2 relaxation rates of the different PLGA fibers over time. c) Comparison of R2 values between SPION‐labeled PLGA fibers and the unlabeled control at the end of the experiment. d) R2 color maps superimposed on T2 weighted images of SPION‐labeled PLGA fibers combined with the PVDF tubular scaffold (top) and of unlabeled controls (bottom) in PBS. e) R2 relaxation rates of 0.2% SPION‐labeled PLGA fibers in comparison to the unlabeled control. f) R2 values of 0.2% SPION‐labeled PLGA fibers decrease as a result of fibers degradation. g) T2 weighted MRI images of the complete TEVGs with SPION‐labeled (top) and unlabeled PLGA fibers (bottom), with corresponding superimposed R2 color map. h,i) R2 relaxometry confirms the degradation of 0.2% SPION‐labeled PLGA fibers in comparison to the unlabeled controls and their faster degradation in the presence of SMCs and ECs. All values were obtained in triplicates: mean ± SD; one‐way ANOVA b,e,h) and *t*‐test c,f,i) with Tukey post hoc correction test were applied; ns > 0.05, ^*^ *p* < 0.05, ^**^ *p* < 0.01, ^***^ *p* < 0.001, and ^****^ *p* < 0.0001.

After confirming the MRI visibility of the SPION‐labeled PLGA fibers, the degradation rate of the copolymer was evaluated. The R2 values of SPION‐labeled PLGA multilayer sheets decreased over time, which can be explained by the release of the SPION from the swollen and degrading fibers (**Figure** **3**a–c; R2 values for control at week 1: (3.6 ± 0.7) s^−1^ versus week 11: (2.3 ± 0.1) s^−1^; 0.2% SPION‐labeled PLGA fibers at week 1: (11.3 ± 8.5) s^−1^ versus week (11: 4.7 ± 0.8) s^−1^; 0.4% SPION‐labeled PLGA fibers at week 1: (21.2 ± 4.0) s^−1^ versus week 11: (5.2 ± 0.5) s^−1^).^[^ ^17^ ^]^

Next, the SPION‐labeled PLGA fibers were investigated in combination with the PVDF tubular structure and incubated in PBS. In compliance with the electrospinning procedure, only the 0.2% SPION concentration was used in this and subsequent studies to avoid clustering of the iron‐oxide nanoparticles at high concentrations.^[^ ^18^ ^]^The change in the relaxation rates (Figure 3e–f), reflecting the degradation of the fibers, was better depicted for the SPION‐labeled PLGA fibers in comparison to the unlabeled control ones (R2 values for unlabeled control at week 1: (2.2 ± 0.1) s−1 versus week 3: (1.7 ± 0.1) s−1; 0.2% SPION‐labeled PLGA fibers at week 1: (7.3 ± 0.6) s−1 versus week 3: (1.9 ± 0.1) s−1). Interestingly, the incubation of the samples in an aqueous environment, such as PBS in comparison to the stiffer fibrin gel used in the previous experimental setup, might have accelerated the degradation rate of the fibers, in this case occurring after 3 weeks compared to 11 weeks observed in the previous study.

The final experiment on the degradation of SPION‐labeled PLGA fibers was performed using complete biohybrid vascular scaffolds (Figure 3g). For this purpose, the textile scaffold consisting of a warp‐knitted PVDF tubular structure coated with SPION‐labeled PLGA fibers was embedded in fibrin gel containing SMCs and ECs. In contrast to the unlabeled control, the degradation of the SPION‐labeled PLGA fibers was again trackable via MRI (R2 values for control at day 1: (4.0 ± 0.3) s^−1^ versus day 7: (3.0 ± 0.9) s^−1^; 0.2% SPION‐labeled PLGA fibers at day 1: (6.2 ± 0.7) s^−1^ versus day 7: (3.5 ± 0.1) s^−1^). The degradation of the fibers was strongly accelerated and occurred in only 7 days (Figure 3h–i). Furthermore, R2 analyses also displayed a slight increase in the medium relaxation values corresponding to the decrease of the R2 values of the SPION‐labeled PLGA fibers, indicating the release of the SPION from the fibers and their following accumulation in the circulating medium (Figure S1, Supporting Information). The faster degradation of PLGA fibers may be explained by the presence of cells. Due to ATP production and oxidative phosphorylation in the mitochondria of cells, the extracellular pH (normally 7.4) can locally become acidic and range between 5.7 and 6.1 during the early stages of wound healing.^[^ ^19^ ^]^ The acidic pH triggers PLGA degradation by ester hydrolysis in acidic conditions.^[^ ^20^ ^]^ This also leads to an enhanced accumulation of PLGA degradation products (lactic and glycolic acid), which further enhances and accelerates the drop in pH and, thus, the degradation process. Furthermore, PLGA degradation was additionally investigated via histological staining after 1, 3, and 7 days of bioreactor cultivation of TEVGs. H&E staining showed the gradual disappearing of the PLGA fiber network and filling of the spaces by cells and new ECM components (Figure S2, Supporting Information).^[^ ^21^, ^22^ ^]^

**Figure 4:**
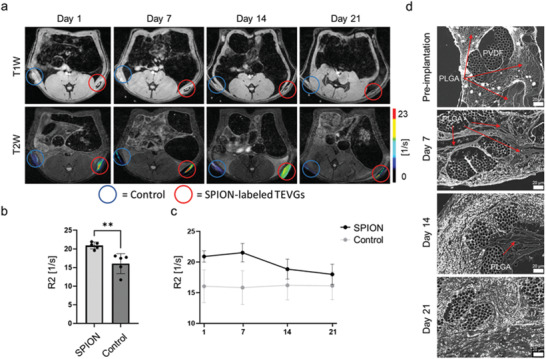
In vivo longitudinal monitoring of SPION‐labeled PLGA degradation. a) T1‐ and T2‐weighted MRI images of subcutaneously implanted SPION‐labeled TEVGs (red circles) and unlabeled controls (blue circles) for 21 days. R2 color maps of both samples superimposed on the gray scale T2‐weighted images highlight the PLGA fibers degradation over time. b) Comparison of the R2 relaxation rate of SPION‐labeled TEVGs and unlabeled controls at day. c) R2 relaxation rate of SPION‐labeled and unlabeled TEVGs over 21 days. d) Ex vivo histological evaluation of explanted TEVGs via H&E staining (shown in gray scale). All values were obtained in triplicates: mean ± SD; *t*‐test b) and one‐way ANOVA c) and with Tukey post hoc correction test were applied; ns > 0.05, ^*^ *p* < 0.05, ^**^ *p* < 0.01, ^***^ *p* < 0.001, and ^****^ *p* < 0.0001.

As an initial proof‐of‐concept for the in vivo visibility and longitudinal monitorability of our vascular grafts, both SPION‐labeled TEVGs and unlabeled control were subcutaneously implanted on the left and right side of the dorsum of Lewis male rats. As shown in **Figure** **4**a, animals were longitudinally monitored for 21 days via MRI. The R2 relaxometry of SPION‐labeled TEVGs showed significantly higher values in comparison to unlabeled controls, indicating a significant improvement in TEVGs detectability due to SPION incorporation (Figure 4b; R2 values for control: (16.1 ± 2.4) s^−1^; SPION‐labeled PLGA fibers: (20.9 ± 0.8) s^−1^). Furthermore, decreases in relaxation rate values during the 21 days of MRI evaluation indicated PLGA fibers’ degradation and the corresponding release of SPION (Figure 4c; R2 values for control at day 1: (16.05 ± 2.4) s^−1^ versus day 21: (16.12 ± 1.8) s^−1^; R2 values for SPION‐labeled PLGA fibers at day 1: (20.9 ± 0.8) s^−1^ versus day 21: 17.9 ± 1.3). Ex vivo H&E staining confirmed the gradual infiltration of cells within the vascular grafts together with the concomitant degradation of the PLGA fibers and their replacement by new ECM (Figure 4d).

### 2.2 Molecular MRI Depicts the Extracellular Matrix

The main mechanical properties of blood vessels are provided by two major ECM components, i.e., collagen and elastin.^[^ ^23^ ^]^ Collagen sponge‐matrix guarantees the tensile stiffness and is required to sustain high blood pressure and to prevent rupture. Elastin confers tissue elasticity, i.e., dynamic stretching under pressure and recoiling to its original configuration.^[^ ^24^, ^25^ ^]^

**Figure 5:**
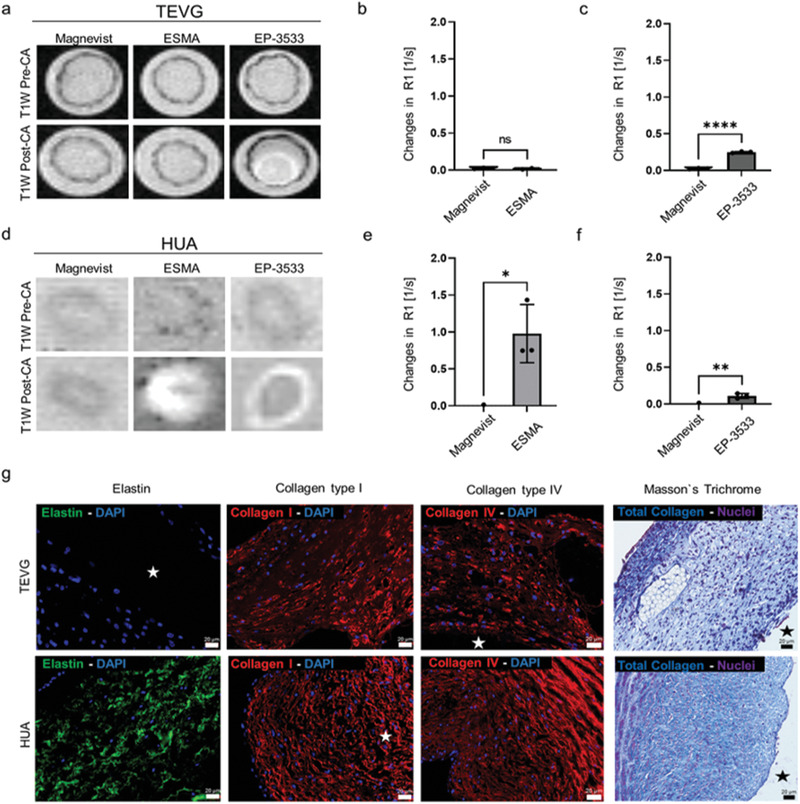
MRI investigation of ECM deposition in TEVGs and HUA. a) T1 weighted MRI images of TEVGs before (top) and after injection (bottom) of ESMA, EP‐3533, and Magnevist. b) Changes in R1 values were obtained by subtracting the pre‐ from the post‐injection values. R1 relaxometry of TEVGs do not markedly change after injection of ESMA. c) Compared to Magnevist, R1 values of TEVGs significantly change after injection of EP‐3533. d) MRI images of HUA (positive control) before (top) and after (bottom) injection of ESMA, EP‐3533, and Magnevist. e,f) R1 relaxometry of HUA after ESMA and EP‐3533 injection indicate the specificity of the probes to their targets. g) Representative histological images of TEVGs (top) and HUA (bottom) showing elastin (green), collagen type I (red), collagen type IV (red) by immunofluorescence, and total collagen (blue) via Masson's trichrome staining. No elastin deposition, but an abundant deposition of collagen (including type I and IV) is detectable in TEVGs. All values were obtained in triplicates: mean ± SD; *t*‐test with Tukey post hoc correction test was applied; ns > 0.05, ^*^ *p* < 0.05, ^**^ *p* < 0.01, ^***^ *p* < 0.001, and ^****^ *p* < 0.0001. The star indicates TEVGs’ and HUA's lumen.

In our TEVGs, ECM remodeling coincides with the PLGA fiber degradation and was characterized using elastin‐targeted (ESMA) and collagen‐type‐I‐targeted (EP‐3533) molecular MRI probes. Both probes have already been extensively characterized.^[^ ^26^, ^27^, ^28^ ^]^ Untargeted Gd‐DTPA (Magnevist, Bayer, Germany) and human umbilical arteries (HUA), which are rich in collagen and elastin, were used as a negative and as a positive control, respectively (**Figure** **5**a–d). Interestingly, the quantitative R1 analyses showed almost no ESMA accumulation in TEVGs (R1 precontrast agents: (0.4± 0.1) s^−1^; R1 post‐ESMA: (0.4 ± 0.1) s^−1^; R1 post‐Magnevist: (0.4 ± 0.1) s^−1^) indicating that no elastin fibers were produced and deposited during the 14 days of bioreactor conditioning and maturation of the vascular grafts (Figure 5b). Furthermore, the lack of elastin fibers deposition within our TEVGs was confirmed via immunofluorescence histology and microscopy (Figure 5g). The regulation of elastin molecular precursor synthesis and the formation of elastic fibers within a 3D fibrin culture remains unclear. Niklason et al. reported only little elastin deposition suggesting that the accumulation of the degradation products of their TEVGs might have impaired ECM deposition.^[^ ^29^ ^]^ In contrast, Jockenhoevel et al. confirmed the presence of elastin within their biohybrid vascular grafts, arguing for a balanced use of fibrin gel and ascorbate for boosting elastin production and deposition.^[^ ^30^ ^]^ In support of the latter, Long and Tranquillo also showed an enhanced elastin production by SMCs, when seeded in fibrin gels, suggesting a positive influence of fibrin on the production of elastin fibers.^[^ ^23^ ^]^ A further strategy to overcome the lack of elastin production was suggested by L'Heureux and colleagues who used fibroblasts instead of SMCs.^[^ ^31^ ^]^ Alternatively, also the employment of bone marrow‐derived smooth muscle progenitor cells might increase elastin deposition.^[^ ^32^ ^]^ Additionally, it is possible to directly incorporate elastin as a textile component by electrospinning or as well by introducing elastin‐like recombinamers into the molding gel.^[^ ^33^, ^34^, ^35^, ^36^ ^]^

In conclusion, the regulation of elastin production, as an important structural element in TEVGs, is still an issue of discussion, and our imaging approach opens an attractive opportunity to study this process longitudinally in intact TEVGs.

In contrast to the lack of elastin in our TEVGs, a hyperintense signal was observed for the collagen type I binding probe EP‐3533 (Figure 5c) and confirmed by T1 relaxometry (R1 precontrast agents: (0.4± 0.1) s^−1^; R1 post‐EP‐3533: (0.6 ± 0.1) s^−1^; R1 post‐Magnevist: (0.4 ± 0.1) s^−1^). Furthermore, histological analyses were performed to investigate the presence of collagen type I and type IV by immunofluorescence, and total collagen via Masson's trichrome staining. Immunofluorescence microscopy confirmed the abundant deposition of collagen fibers within our TEVGs, which visually appeared comparable to collagen deposition in HUA. Moreover, we also investigated early collagen type I deposition by histology and confirmed its presence after 3 days of bioreactor maturation of our TEVGs (Figure S3, Supporting Information). These findings are in line with previous studies, demonstrating enhanced collagen production by SMCs when TEVGs’ in vitro maturation is carried out under dynamic conditions.^[^ ^37^, ^38^ ^]^ MR imaging was also performed on HUA samples (Figure 5e–f) to confirm the validity and functionality of our imaging approach. For both ESMA and EP‐3533, a high and statistically significant increase in signal intensities was found, which was absent for Magnevist. The signal changes in the MR images could be confirmed by relaxometry (R1 precontrast agents: (0.5 ± 0.003) s^−1^; R1 post‐ESMA: (1.5 ± 0.04) s^−1^; R1 post‐EP‐3533: (0.6 ± 0.03) s^−1^; R1 post‐Magnevist: (0.5 ± 0.08) s^−1^). Furthermore, to investigate the MRI sensitivity to the evaluated targeted probes, we introduced known amounts of collagen type I and elastin in the fibrin molding gel, respectively. The ESMA and EP‐3533 R1 values correlate linearly with elastin and collagen type I amounts within the employed the acellularized scaffold (Figure S4, Supporting Information). Our data are supported by previous studies, in which the linear correlation between R1 values and ESMA or EP‐3533 fraction bound to elastin or collagen type I has already been shown, e.g., by ICP‐MS, by Western blot, and by staining and Gd analysis.^[^ ^27^, ^39^, ^40^ ^]^ Consequently, molecular MRI proved to be an efficient tool to characterize the deposition and remodeling of the two main ECM components in TEVGs in vitro. Thus, the highly sensitive evaluation of ECM deposition during the in vitro conditioning of the vascular prostheses might represent a fundamental step forward to predict and ameliorate the host's outcomes upon in vivo implantation.

### 2.3 Molecular Ultrasound Imaging Assesses Endothelial Integrity and Inflammation

*α*_v_ *β*_3_ integrin is an important marker of inflammation and thrombus formation. This integrin directly interacts with *α*_IIb_ *β*_3_ integrins on platelets and plays a role in several pathways leading to hyperplasia of the intima and, thus, stenosis of vascular grafts.^[^ ^39^ ^]^ It has been shown that, especially after the disruption of endothelial coverage, *α*_v_ *β*_3_ expression increases and induces strong SMCs proliferation toward the inner lumen of the vessel.^[^ ^40^, ^41^, ^42^ ^]^

**Figure 6:**
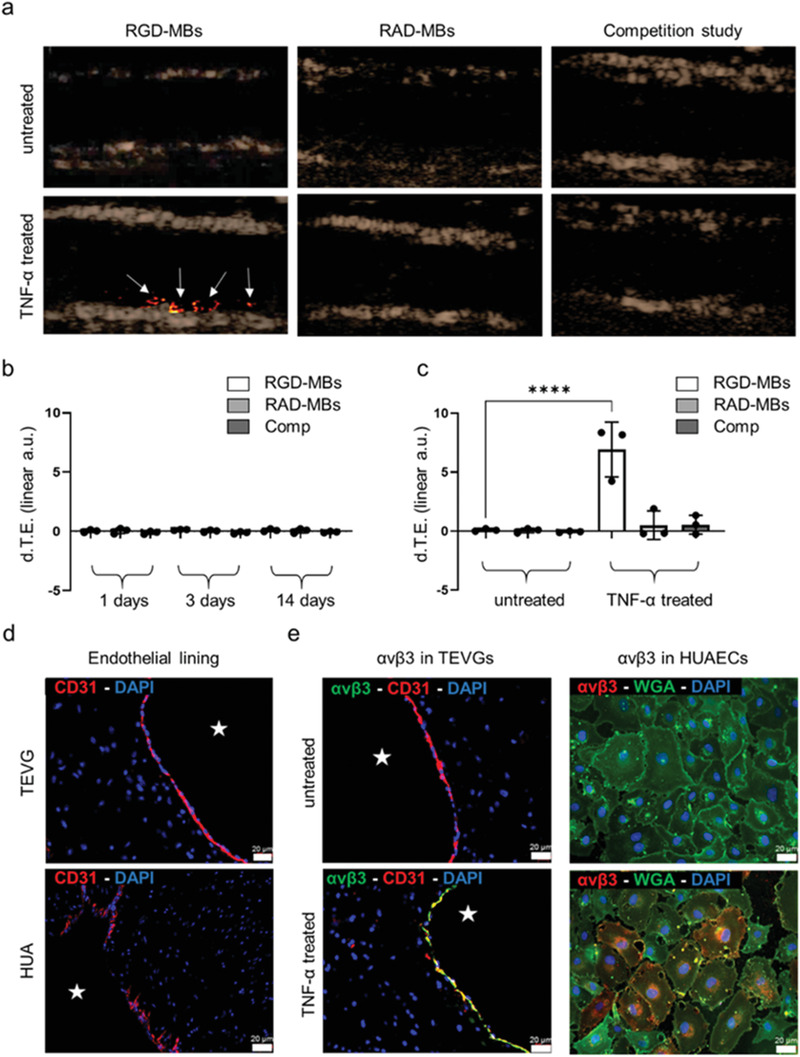
Molecular US imaging of *α*_v_ *β*_3_ integrin expression. a) Contrast mode ultrasound images are shown with a color‐coded overlay of the d.T.E., the latter indicating bound MBs (white arrows). While there is hardly any c[RGDfK]‐MB binding in untreated TEVGs (top), TNF‐*α* treatment of TEVGs results in a strong (bottom) c[RGDfK]‐MB attachment to the inner lumen of the TEVGs. In both, untreated and TNF‐*α* treated TEVGs no significant binding was observed after the injection of control c[RADfK]‐MB, and the injection of c[RGDfK]‐MB after blocking integrins with free c[RGDfK], respectively. b) Quantitative analysis of molecular US imaging data acquired at 1, 3, and 14 days of TEVGs’ bioreactor conditioning. c) Comparison between c[RGDfK]‐MB binding in TNF‐*α* treated and untreated TEVGs. d) Immunofluorescence histology of ECs (red) in TEVGs and HUA to evaluate the endothelial lining integrity and continuity. e) Immunofluorescence images of *α*_v_ *β*_3_ integrin expression in untreated (top) and TNF‐*α* treated (bottom) TEVGs and HUAECs. In TEVGs, histology shows colocalization of *α*_v_ *β*_3_ (red) with CD31 (green) only after TNF‐*α* injection. The basal level of *α*_v_ *β*_3_ integrin expression is investigated in HUAECs. Only after TNF‐*α* treatment, HUAECs show colocalization of *α*_v_ *β*_3_ (red) with wheat germ agglutinin (WGA) (green), indicating the cells’ nonactive phenotype. All values were obtained in triplicates: mean ± SD; *t*‐test with Tukey post hoc correction test was applied; ns > 0.05, ^*^ *p* < 0.05, ^**^ *p* < 0.01, ^***^ *p* < 0.001, and ^****^ *p* < 0.0001. The star indicates TEVGs’ and HUA's lumen.

Therefore, *α*_v_ *β*_3_ integrin expression is an excellent marker of endothelial dysfunction in our TEVGs. We decided to assess *α*_v_ *β*_3_ integrin expression by molecular US imaging as it is noninvasive and easily applicable in vitro and in vivo. Furthermore, even if the entire endothelial layer is destroyed, luminal availability of *α*_v_ *β*_3_ integrin is still preserved by activated SMCs. Thus, *α*_v_ *β*_3_ integrin expression would still indicate failures in the endothelialization of TEVGs. Molecular US imaging in TEVGs showed comparable MB binding signal between c[RGDfK]‐MB and c[RADfK]‐control MBs (**Figure** **6**a), indicating no integrin expression. The competitive binding experiment, performed at all three observation time points confirmed this result (Figure 6b). MB binding was investigated and acquired in nonlinear contrast mode (NLC), which removes all the linear response from tissue signal from the quantification process and allows a highly accurate quantification of molecular biomarkers. The final values obtained after quantification were expressed as differential targeted enhancement (d.T.E.). The linear correlation between US signals and MBs concentrations was also evaluated in phantoms reaching a correlation coefficient of R2 = 0.9905 (Figure S5, Supporting Information). The lack of *α*_v_ *β*_3_ integrin expression in our TEVGs might be due to the absence of the complement system precursors and the different types of immune system cells. Furthermore, in line with our findings, it has been shown that *α*_v_ *β*_3_ integrin is minimally expressed on resting endothelium, and its upregulation is induced by mechanical endothelial denudation, shear stress, growth factors, and cytokines.^[^ ^43^ ^]^

Therefore, TNF‐*α*, one of the major inflammatory cytokines, was used to increase *α*_v_ *β*_3_ integrin expression in our TEVGs to mimic a situation of endothelial dysfunction.^[^ ^44^ ^]^ TNF‐*α* treated TEVGs showed an increased c[RGDfK]‐MB binding in comparison to c[RADfK]‐control MBs and a decreased binging after competitive blocking of *α*_v_ *β*_3_ integrins (Figure 6a,c). Furthermore, the preactivation of cells with TNF‐*α* led to a 70‐fold increase in c[RGDfK]‐MBs binding compared to the untreated TEVGs (d.T.E. for c[RGDfK]‐MB in untreated TEVGs: 0.1 ± 0.1 versus in TNF‐*α* treated TEVGs: 6.9 ± 2.3).

Subsequently, immunofluorescence analyses were performed to evaluate the endothelial coverage and *α*_v_ *β*_3_ integrin expression in TEVGs (Figure 6d–e). Histology confirmed the presence of a continuous endothelial layer and the *α*_v_ *β*_3_ integrin expression by ECs only in TNF‐*α* treated TEVGs (Figure 6e). To further investigate the basal expression level of *α*_v_ *β*_3_ integrin, histological analyses were performed on 2D cultured human umbilical artery endothelial cells (HUAECs), shown in Figure 6e, and on HUA, serving as positive controls (Figure S6, Supporting Information). In line with the TEVGs, the 2D cultured HUAECs did not express *α*_v_ *β*_3_ integrins prior to TNF‐*α* activation. In contrast, HUA samples physiologically expressed *α*_v_ *β*_3_ integrins, even in absence of ongoing inflammatory reactions. Subsequently, we can conclude that the bioreactor design that kept shear stress low as well as the dynamic flow conditions applied for the in vitro maturation of our TEVGs’ might have caused only shallow levels of distress to the ECs being not sufficient to stimulate *α*_v_ *β*_3_ integrin expression. Moreover, there might be a possible correlation between the lack of integrins and elastin in our TEVGs. It is well known that the integrin system, especially *α*_v_ *β*_3_ integrins, physiologically influences the cytoskeleton remodeling through a complex interplay of signaling pathways.^[^ ^45^, ^46^, ^47^ ^]^ The cytoskeleton remodeling has also been achieved in vitro by repeated stretching cycles and, thus, stressing of SMCs resulting in higher production and deposition of elastin.^[^ ^48^ ^]^ Therefore, several studies on the matter seem to suggest that the absence of integrins, which modulate the stretching of cellular components and ECM formation in response to physiological pulsatile flow and pressure, might result in impaired ECM deposition, i.e., the lack of elastin.

Consequently, here we show that the employment of MRI and molecularly targeted US efficiently enables the noninvasive longitudinal monitoring of scaffold resorption, ECM remodeling, and endothelial integrity. Evaluating these aspects before and after the implantation of vascular grafts is fundamental to ensure their integrity and functionality. The application of MRI in tissue engineering has several advantages, such as excellent imaging penetration depth as well as superior soft‐tissue contrast, providing anatomical, functional, and cellular information. Furthermore, MRI is an imaging technique routinely applied for clinical investigations. Here, the direct labeling of the textile scaffold with MRI contrast agents, such as SPIONs, holds great translational potential as five SPION agents are already clinically approved.^[^ ^49^ ^]^ Alternatively, labeling scaffolds with ^19^F MRI might represent a valuable addition to ^1^H MRI. For example, ^19^F‐labeled compounds can be used to label cells and synthetic polymers with a sufficient number of highly mobile ^19^F atoms can be applied as parts of the scaffold material.^[^ ^50^, ^51^ ^]^

Gadolinium‐based MR contrast agents are the most used ones in clinics. However, there are always concerns about the release of gadolinium from the chelate complexes and potential resulting adverse effects.^[^ ^52^ ^]^ As the persistence of these agents in the body correlates with gadolinium release, the use of these molecular MRI probes is considered particularly critical. Nevertheless, new MRI probes were designed to detect mesenchymal stem cell activity, ECM deposition, and remodeling via matrix metalloproteinases, showing promising results.^[^ ^26^, ^27^, ^53^, ^54^ ^]^ Concerning the agents used in this study, we can say that the pharmacokinetic properties and Gd‐release kinetics of ESMA and EP‐3533 were already tested and appear acceptable.^[^ ^55^, ^56^ ^]^ Furthermore, even if the agents cannot be approved for MRI, there is still the option to entrap radiometals in the chelate complexes and use them for SPECT or PET imaging as an attractive clinical alternative.

X‐ray and CT have been extensively used to characterize and assess tissue‐engineered scaffold properties as porosity and integrity.^[^ ^57^, ^58^, ^59^ ^]^ For instance, CT imaging might be applied to monitor the degradation of acellularized vascular graft scaffolds by assessing the changes in volume and density, or combined with other imaging modalities, such as positron emission tomography (PET), to localize and quantify vascular inflammation.^[^ ^4^, ^58^ ^]^ Nonetheless, CT does not represent an ideal tool for the noninvasive imaging of vascular implants compared with MRI, mainly because of lower tissue contrast and radiation exposure.^[^ ^60^ ^]^

US is another valuable imaging tool to monitor vascular grafts’ functionality in vitro and in vivo. It provides excellent temporal resolution and reasonable tissue penetration. US can be used to assess vessel wall integrity and patency of vascular implants or to evaluate the endothelial coverage, atherosclerotic plaque formation, and inflammatory reaction through the employment of targeted contrast agents, the latter being already clinically employed or currently under clinical trial.^[^ ^14^, ^61^, ^62^, ^63^ ^]^

In addition to *α*_v_ *β*_3_ integrins imaging, which was carried out in our study, other targeting ligands might be suitable for the detection of endothelial lining integrity or distress. For instance, major endothelial markers of inflammation, such as P‐selectin, von Willebrand factor (VWF), vascular endothelial growth factor (VEGF) receptors, vascular cell adhesion molecule 1 (VCAM‐1), and intercellular adhesion molecule 1 (ICAM‐1), have been extensively explored during the investigation of cardiovascular diseases as early detection of atherosclerotic plaque.^[^ ^64^, ^65^, ^66^, ^67^, ^68^, ^69^, ^70^ ^]^ Furthermore, Curaj et al. showed the high sensitivity of junctional adhesion molecule A (JAM‐A) functionalized MB for the US detection of transient endothelial activation, which could be an important early indicator of endothelial distress in the TEVGs.^[^ ^71^ ^]^

## 3 Conclusion

In this manuscript, we present a comprehensive imaging approach to characterize TEVGs noninvasively and nondestructively. This includes the visualization of textile scaffold components, ECM deposition, and the assessment of endothelial integrity. In detail, we propose the incorporation of SPION into PLGA fibers to efficiently monitor their degradation by MRI both in vitro and in vivo. Indeed, the subcutaneous implantation of our TEVGs into a rat model provides an initial proof‐of‐concept for the in vivo detection and longitudinal evaluation of our SPION‐labeled PLGA fibers. Furthermore, we show that using the same imaging modality, both elastin and collagen type I deposition can be effectively assessed providing valuable insights into the maturation and remodeling of the TEVGs. We also demonstrate that molecularly targeted US is an efficient tool for the assessment of endothelial dysfunction that may result in hyperplasia of the intima, thrombus formation, and loss of patency of the vascular grafts. Consequently, our imaging approach might strongly support quality control in the critical transition phase between late in vitro bioreactor maturation and early in vivo implantation of cardiovascular implants and thus, foster their clinical translation.

## 4 Experimental Section

### Synthesis of Oleic Acid‐Coated SPION

Oleic acid‐coated SPION were synthesized by thermal decomposition according to a previously established protocol.^[^ ^72^, ^73^ ^]^ Purified iron (III) oleate precursor (8 mmol) and oleic acid (58.4 mmol) were dissolved in 1‐octadecene (50 mL) in a three‐neck round‐bottom flask insulated with glass wool and aluminum foil. The ratio of oleic acid to precursor was kept constant at 7:3. Then, the mixture was heated to 100 °C and maintained at this temperature without reflux to evaporate residual water. After degassing and fitting a reflux cooler, the reaction mixture was heated further to 340 °C with a heating rate of 3 °C min^−1^ under argon atmosphere and aged at the same temperature for 3 h. After cooling down the sample to room temperature, the sample was transferred to 50 mL Falcon tubes, and acetone was added. To assist the deposition of nanoparticles and to increase the extraction of impurities from the solvent, the sample was kept at 50 °C in a water bath for 5 min and then sonicated for 5 min. The sample was centrifuged, and the supernatant was discarded. Then, the nanoparticles were dispersed in a mixture of hexane and methanol with a ratio of 1:6. The mixture was centrifuged, and the washing of the sample was repeated three more times with hexane and methanol. In the end, the washed nanoparticles were dispersed in hexane. After the addition of one drop of oleic acid, the final sample was stored at 4 °C until further use.

### PVDF Mesh Production

By using PVDF (Lenzing Plastics GmbH & Co. KG, Lenzing, Austria) multifilament fibers (150 dtex, 48 filaments), three different tubular graft structures were produced using a double‐bar raschel warp‐knitting machine (Karl Meyer Holding GmbH & Co. KG, Obertshausen, Germany). The number of fibers was altered (4, 6, and 10 fibers) to achieve different inner diameters of the resulting round knitted mesh structures. The warp‐knitting process parameters are stated in **Table** **1**.

**Table 1:** Process parameters of the warp‐knitting process of the tubular PVDF grafts

Graft area	Warp‐knitting chain notation	Effective arcs per cm (EAC) value
Front side	2/4‐2‐2‐2‐0‐2‐2‐2‐4‐2‐2‐2‐0‐2/2//	EAC 6
Back side	2/2‐2‐0‐2‐2‐2‐4‐2‐2‐2‐0‐2‐2‐2/4//	EAC 6
Brim fibers	0‐2‐2‐2‐2‐2‐0‐2‐0‐2‐2‐2‐2‐2‐0/2// 0‐0‐2‐0‐2‐0‐0‐0‐0‐0‐2‐0‐2‐0‐0‐0//	EAC 6

### Electrospinning of SPION‐Labeled PLGA Fibers

The warp‐knitted grafts were coated by electrospun PLGA (Purasorb PLG 8523, Corbion Purac Gorinchem, Netherlands) fibers labeled with SPION. All spinning procedures were performed using a coaxial spinning head (Bioinicia SL, Paterna, Spain) with two coaxial arranged steel capillaries with inner diameters of 1400 µm (shell capillary) and 580 µm (core capillary). SPION‐labeled PLGA fibers were spun at a voltage of +22 kV (emitter) and −20 kV (collector). The electrospinning of the fibers was performed at 25 °C and 30% of humidity. Three different spinning solutions were produced, including 6% (w/w) PLGA (shell solution), 6% (w/w) PLGA labeled with 0.2% (w/w) SPION (core solution), and 6% (w/w) PLGA labeled with 0.4% (w/w) SPION (core solution) dissolved in methanol (MeOH, neoLab Migge Laborbedarf – Vertriebs GmbH, Heidelberg, Germany) and chloroform (CHCl_3_, Carl Roth GmbH & Co. KG, Karlsruhe, Germany). Each spinning solution was prepared (10 mL) and stirred for 24 h until the PLGA was completely dissolved. The SPION solution was added to the core solution shortly before the execution of the electrospinning process and mixed gently until reaching a homogenous solution. For coating of the PVDF grafts, the core of the coaxial spinning head was loaded with the core solution and the shell solution was loaded into the shell of the coaxial spinning head, respectively. For each coating procedure, a PVDF graft (30 cm) was put on a cylindrical collector (tip to collector distance of 20 cm). The spinning head was moved continuously along the entire length of the graft during the spinning process with a speed of 30 mm s^−1^. Coating was performed on different samples for 10 or 30 min. The flow rates of the core and shell solution were set to 0.5 and 1 mL h^−1^ during the spinning process.

### Biohybrid Tissue‐Engineered Vascular Graft Production and Bioreactor Conditioning

The molding of the vascular graft was performed inside a silicon tube (6.4 mm inner diameter) (Carl Roth GmbH, Germany). A metal rod (3 mm diameter) surrounded by the textile scaffold was inserted as a core. Subsequently, the matrix solution was injected into the molding cavity. The matrix solution consisted of fibrinogen (10 mg mL^−1^) (Calbiochem, Darmstadt, Germany), thrombin (40 U mL^−1^) (Sigma, Steinheim, Germany), tris buffer, CaCl₂ (50 ×10^−3^ m) (Sigma, Steinheim, Germany) and human arterial smooth muscle cells (10^7^ cells mL^−1^). The metal rod was removed after the molding, and human arterial endothelial cells (10^6^ per cm graft) were injected into TEVG's lumen for the endothelialization process.

**Figure 7:**
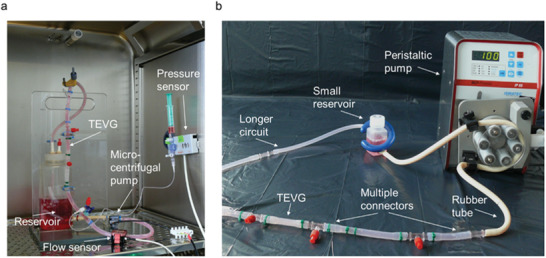
Visual display of the custom‐made bioreactor modifications. a) The closed bioreactor circuit was connected to a 500 mL medium reservoir, a micro‐centrifugal pump, and to pressure and flow sensors. b) Later, the bioreactor circuit was equipped with longer silicon and rubber tubes, a small medium reservoir fitting into the 7T MRI, and a peristaltic pump so that TEVGs were easily insertable and removable during the imaging procedure.

Subsequently, TEVGs were conditioned in a bioreactor system consisting of a silicon tube closed circuit containing the vascular graft and a medium reservoir (500 mL) (**Figure** **7**a). The dynamic flow of the medium within the system was provided by a micro‐centrifugal pump (Rs Pro, Corby, United Kingdom) and the generated flow and pressure were monitored by a flow sensor (Em‐tec GmbH, Finning, Germany) and a pressure sensor (Codan GmbH, Lensahn, Germany). To enable multimodal imaging at the same time, the bioreactor was modified (Figure 7b). The silicon tube system was elongated by multiple connectors and rubber tubes (Saint‐Gobain, Herzogenrath, Germany), the micro‐centrifugal pump was replaced by a peristaltic pump (MCP Process, Ismatec, Cole Parmer GmbH, Germany), and the medium reservoir was substituted with a smaller bottle (50 mL) to fit into the 7T MRI.

### Enhancing the PLGA Fibers Detectability by SPION Labeling

MRI phantoms were produced by embedding two different concentrations of SPION‐labeled PLGA fibers (0.2% and 0.4%) in 10% gelatin (w/v) and compared to unlabeled ones used as a negative control. Subsequently, both 0.2% and 0.4% of SPION‐labeled PLGA fibers were analyzed in combination with the PVDF tubular structure. Moreover, the electrospinning time for the production of the PLGA coating layer upon the PVDF meshes was also investigated to assess whether longer coating time might influence the MRI visibility of the fibers. Next, the phantoms were measured in a Bruker BioSpec 70/20 USR 7T MRI scanner (Bruker BioSpin GmbH, Germany) using a ^1^H transmit‐receive volume coil with active detuning with an inner diameter of 82 mm and a bore length of 112 mm.

Initially, shimming based on B0 field mapping was performed to reduce the nonlinear image distortions due to the inhomogeneities of the static magnetic field (B0).

Transverse relaxation times (T2) were measured using a 2D multi‐slice, multi‐echo spin‐echo (MSME) sequence with a 90° excitation pulse followed by a train of equally spaced 180° refocusing pulses [repetition time (TR) = 2000 ms; echo time (TE) = 20 ms; echo spacing = 20 ms; echo images = 30; number of averages = 4; matrix size = 150 × 150; FOV = (35 × 20) mm^2^; slice thickness = 1 mm].

T2* relaxation times were measured using a multiple gradient echo (MGE) sequence [TR = 800 ms; TE = 4.5 ms; echo spacing = 5.5 ms; echo images = 30; number of averages = 6; flip angle = 50°; matrix size = 150 × 150; FOV = (35 × 20) mm^2^; slice thickness = 1 mm].

T2 and T2* relaxation times and the respective R2 and R2* relaxation rates were calculated by fitting an exponential curve to the signal amplitudes as a function of the echo time (TE) for each segmented region of interest (ROI) obtained by using the Imalytics Preclinical Software (Gremse‐IT GmbH, Aachen, Germany). The fitting of the exponential curve was performed by using Equation (1)

(1) $$M\;=M_{0}\;\;e^{-\frac{\mathrm{TE}}{T2}}+C$$

in which an offset (*C*) was included to account for a signal plateau created by noise or a component with slow signal decay. R2 and R2* were calculated as the inverse of T2 and T2*, respectively.

Moreover, T2‐ and T2*‐ weighted images were acquired using a T2‐weighted fast spin echo sequence [TR = 3878 ms; TE = 80 ms; echo spacing = 40 ms; acceleration factor = 4; matrix size = 360 × 150; FOV = (35 × 20) mm^2^; slice thickness = 1 mm] and a T2*‐weighted gradient echo sequence [TR = 907 ms; TE = 30 ms; flip angle = 18°; matrix size = 360 × 150; FOV = (35 × 20) mm^2^; slice thickness = 1 mm], respectively.

### Longitudinal MRI Monitoring of the PLGA Fiber Degradation Rate

SPION‐labeled and unlabeled control fibers were embedded in fibrin phantoms. The fibrin gel consisted of fibrinogen (1 mL) (Merck KGaA, Darmstadt, Germany) dissolved in TBS (10 mg mL^−1^), thrombin (150 µL) (Merck KGaA, Darmstadt, Germany), aprotinin (20 U mL^−1^) (Abcam, Berlin, Germany), and CaCl_2_ (150 µL) (Sigma, Steinheim, Germany). Fibrin phantoms were imaged using the 7T MRI scanner for a total duration of 11 weeks. The samples were incubated at 37 °C for the first 8 weeks and at 70 °C until the end of the experiment to accelerate the degradation of the PLGA.

In addition, PLGA degradation was monitored in combination with the PVDF tubular structures. For this purpose, PVDF filaments coated with PLGA were incubated in PBS at 37 °C for 3 weeks. The same MRI sequences as for the gelatin phantoms were employed for the acquisition of T2‐/T2*‐weighted images and T2/T2* relaxation times. Colormaps were obtained using Imalytics Preclinical Software (Gremse‐IT GmbH, Aachen, Germany) and by superimposition of the calculated R2 colormap onto the corresponding T2‐weighted image.

### Assessment of PLGA Degradation in Complete TEVGs

TEVGs were longitudinally studied by 7T MRI over a period of 7 days to investigate whether the degradation of SPION‐labeled PLGA fibers was influenced by the presence of cells. TEVGs were monitored immediately after endothelialization of the lumen of the vascular graft as well as after 3 and 7 days, respectively.

T2 relaxometry was performed using a MSME sequence [TR = 5600 ms; TE = 20 ms; echo spacing = 20 ms; echo images = 30; number of averages = 4; matrix size = 150 × 150; FOV = (35 × 20) mm^2^; slice thickness = 1 mm] and Equation (1) was used for the fitting of the exponential curve. The shown R2 colormaps were calculated and obtained as for the previous studies.

### In Vivo Longitudinal MRI Evaluation of PLGA Degradation in TEVGs

A total of six inbred male Lewis rats (Janvier Labs, Le Genest‐Saint‐Isle, France), aged 12 weeks, were used. The animal experiments were approved by the governmental review committee on animal care (Landesamt für Natur, Umwelt und Verbraucherschutz Nordrhein‐Westfalen, LANUV, NRW). Under general (Isoflurane, 2% in O_2_) and local anesthesia (Ropivacaine 2 mg kg^−1^), each rat underwent a surgical procedure, which consisted of the subcutaneous implantation of 1 cm of SPION‐labeled TEVG on the left side and 1 cm of unlabeled control on the right side of the dorsum. The cells incorporated into both SPION‐labeled and unlabeled TEVGs were obtained by previously sacrificing one Lewis rat, which was used as a cell donor to avoid immunogenicity. On days 1, 7, 14, and 21 post‐implantation, the MRI investigation was performed under isoflurane anesthesia using a 7T MRI equipped with a ^1^H transmit‐receive volume coil with active detuning. One animal each was sacrificed after days 7 and 14, and the remaining three at day 21 to allow further ex vivo histological analyses. Anatomical T2‐ and T1‐weighted images were acquired by using a T2‐weighted fast spin echo sequence [TR = 1300 ms; TE = 25 ms; echo spacing = 8.3 ms; acceleration factor = 8; matrix size = 180 × 180; FOV = (50 × 60) mm^2^; slice thickness = 1 mm] and a T1‐weighted fast spin echo sequence [TR = 200 ms; TE = 2.9 ms; flip angle = 30°; matrix size = 180 × 180; FOV = (50 × 60) mm^2^; slice thickness = 1 mm], respectively. T2 relaxometry was performed using a MSME sequence [TR = 4445 ms; TE = 7 ms; echo spacing = 20 ms; echo images = 40; number of averages = 1; matrix size = 144 × 112; FOV = (50 × 60) mm^2^; slice thickness = 1 mm].

### Molecular MRI of Elastin and Collagen in Tissue‐Engineered Vascular Grafts

To study the production and deposition of the extracellular matrix, ESMA (Lantheus Medical Imaging, North Billerica, Massachusetts, USA) and EP‐3533 (Collagen Medical LLC, Belmont, Massachusetts, USA) were used molecular MRI probes to assess elastin and type I collagen deposition, respectively. Both MRI probes were compared to the nonspecific clinical contrast agent Gd‐DTPA (Magnevist, Bayer, Germany).

TEVGs were measured with 7T MRI after 14 days of bioreactor conditioning. Following the acquisition of T1 relaxation times, the nonspecific contrast agent Gd‐DTPA (0.3 mmol mL^−1^) was injected into the bioreactor with a peristaltic pump (MCP‐Process, Ismatec, Cole‐Parmer GmbH, Germany) at a constant flow rate of 0.5 mL min^−1^ and circulated at room temperature for 1 h, followed by washing with fresh cell medium (Lonza Bioscience, North Carolina, United States) for 1 h. Then, another T1 relaxometry measurement was performed to assess the change in T1 relaxation time. On the same TEVGs, the procedure was repeated for ESMA (0.2 mmol mL^−1^) and EP‐3533 (10 µmol mL^−1^), respectively.

T1 relaxation times were acquired using a fast spin echo sequence with static TE and variable TR [number of T1 Experiments = 6; TR = 5000–275 ms; TE = 12 ms; acceleration factor = 4; echo spacing = 6 ms; number of averages = 1; matrix size = 100 × 100; FOV = (10 × 10) mm^2^; slice thickness = 1 mm]. T1 was calculated by fitting an exponential curve to the signal amplitudes as a function of the TR for each segmented ROI obtained by using the Imalytics Preclinical Software (Gremse‐IT GmbH, Aachen, Germany). The fitting of the exponential curve was performed by using Equation (2)

(2) $$M\;=\;M_{0}\left(1-e^{-\frac{\mathrm{TR}}{T1}}\right)$$

Moreover, T1‐weighted images were acquired using a fast spin echo sequence [TR = 150 ms; TE = 2.9 ms; flip angle = 50°; matrix size = 100 × 100; FOV = (50 × 60) mm^2^; slice thickness = 1 mm] In addition, the three molecular MRI probes were applied on HUA serving as positive controls regarding the presence of elastin and type I collagen. The employment of human samples used throughout this study was performed in compliance with the Ethical Committee approval (278/16).

### PBCA MB Synthesis and Coupling of Peptides

PBCA MBs were synthesized based on a previously described protocol.^[^ ^74^, ^75^ ^]^ Triton X‐100 (3 mL) (Sigma‐Aldrich, Darmstadt, Germany) was dissolved in distilled water (300 mL) via means of a magnetic stirrer. The pH of the solution was decreased to 2.5 by dropwise addition of hydrochloric acid (1 m). Subsequently, butyl cyanoacrylate (BCA) (Special polymers Ltd., Sofia, Bulgaria) (3 mL) was added dropwise to the aqueous solution and homogenized at 10 000 rpm by using an Ultra‐Turrax T‐50 (IKA Werke GmbH & Co. KG, Staufen, Germany) for 1 h. The emulsion was then transferred into Falcon tubes (50 mL), centrifuged at 500 rpm to remove the debris for 20 min, and washed three times with 0.02% Triton X‐100 aqueous solution. The size and concentration of the MBs were determined using a Coulter counter (Multisizer 4e, Beckman Coulter Life Sciences, Indiana, USA). For each measurement, the MBs (2 µL) were dispersed in ISOTON II diluent (20 mL) (Beckman Coulter). Based on a previously established protocol, MBs' surface was functionalized with biotinylated c[RGDfK] and c[RADfK] peptides (Vivitide, Gardner, Massachusetts, USA), the MBs were hydrolyzed with NaOH (0.1 n, pH 11) at room temperature under constant rotation at 40 rpm for 15 min.^[^ ^76^ ^]^ Then, hydrochloric acid (1 m) was used to decrease the pH to 2.5–3. The MBs were centrifuged at 500 rpm for 20 min and washed with 0.02% Triton X‐100 aqueous solution. A second solution of 1‐ethyl‐3‐(3‐dimethylaminopropyl) carbodiimide (10 mg) (Carl Roth GmbH, Karlsruhe, Germany) mixed with triethanolamine (2 µL) (Acros Organics, Schwerte, Germany) was prepared in a glass vial using a magnetic stirrer. MBs were added to this solution and, after 30 s of stirring, streptavidin (1 mg mL^−1^) was added. After stirring at 150 rpm at room temperature for 1 h, the mixture was kept at 4 °C for 12 h. Then, in presence of 0.02% HEPES (4‐(2‐hydroxyethyl)‐1‐piperazineethanesulfonic acid) buffer, the MBs were washed by 3 centrifugation steps at 500 rpm for 20 min. Subsequently, they were mixed with biotinylated c[RGDfK] or c[RADfK] at room temperature under constant rotation at 40 rpm for 15 min, and again washed with 0.02% Triton X‐100 aqueous solution.

### Molecular Ultrasound Imaging of TEVGs

US imaging was performed using a VEVO 3100 ultrasound system equipped with a linear‐array‐MX‐250 transducer (FUJIFILM VisualSonics, Toronto, Ontario, Canada). To enable the specific binding of c[RGDfK]‐functionalized MBs in the TEVGs under flow conditions, the modified bioreactor circuit was placed horizontally on the ultrasound table. Then, the transducer was placed transversally onto the silicone tube containing the TEVGs to obtain sagittal images of the lumen of the graft. The flow rate was maintained constant at 0.5 mL min^−1^ using a peristaltic pump. The accumulation of c[RGDfK]‐functionalized MBs was compared with c[RADfK]‐functionalized ones, the latter serving as negative controls. Furthermore, a competitive binding experiment was performed by injecting free c[RGDfK] prior to the injection of c[RGDfK]‐functionalized MBs in access. c[RADfK]‐functionalized MBs (1 × 10^9 ^MB mL^−1^) were injected into the medium reservoir of the bioreactor circuit, and the inflow of the MBs was imaged in NLC mode at 18 MHz frequency, 4% power, and 500 frames were acquired. The MBs were circulated for 8 min. Subsequently, the medium with the unbound MBs was removed, the TEVGs were washed with sterile DPBS, and fresh medium was added. 500 frames were recorded at 4% power and 18 MHz frequency, and a 100% power destructive pulse was applied after 250 frames to destroy the bound MBs. The same procedure was repeated for the c[RGDfK]‐functionalized MBs and the competitive binding experiment. For quantifying the contrast intensity given by target‐bound MBs, ROI were drawn using the VevoLAB software version 3.2 (FUJIFILM VisualSonics, Toronto, Ontario, Canada). Subsequently, the d.T.E. was calculated by subtracting the signal before and after MB destruction.

### Immunohistochemistry

The samples were fixed in Carnoy's solution at room temperature for 3 h, washed in ethanol for 1 h, dehydrated by using a dehydration device (Leica TP 1020, Wetzlar, Germany) for 12 h, and embedded in paraffin. 5 µm thick paraffin sections were cut using a microtome (Microm HM 430, Thermofisher Scientific, Massachusetts, USA), dried at 37 °C for 12 h, and stored at room temperature. Prior to staining, the samples were deparaffinized by serial dilution of xylol and ethanol. Subsequently, the object slides were placed inside Sequenza Staining racks (Thermofisher Scientific, Massachusetts, USA) to allow the blocking and permeabilization of the samples by using a 0.1% Triton X‐100 aqueous solution containing 5% of normal goat serum (NGS) (Agilent Dako, Santa Clara, California, USA) at room temperature for 1 h. The samples were incubated with the primary antibody at 37 °C for 1 h, washed three times with PBS for 5 min, and incubated with the respective secondary antibody for 1 h. Subsequently, the samples were washed three times with PBS for 5 min, incubated with DAPI (1:500) (Thermofisher Scientific, Massachusetts, USA) for 5 min, washed three times with PBS for 5 min, and mounted with fluorescent mounting medium (Agilent Dako, Santa Clara, California, USA). The primary antibodies used in this study are: monoclonal, anti‐human alpha‐smooth muscle actin (*α*‐SMA) (Sigma‐Aldrich, Darmstadt, Germany); monoclonal, anti‐human CD31 (Sigma‐Aldrich, Darmstadt, Germany); polyclonal, anti‐human type I collagen (Acris Antibodies GmbH, Herford, Germany); polyclonal, anti‐human type IV collagen (Acris Antibodies GmbH, Herford, Germany); polyclonal, anti‐human elastin (Acris Antibodies GmbH, Herford, Germany); monoclonal, anti‐human *α*_v_ *β*_3_ integrin (eBioscience, San Diego, California, USA). The secondary antibodies used throughout this study are anti‐mouse Alexa Fluor 488 (Thermofisher Scientific, Massachusetts, USA), anti‐mouse Alexa Fluor 594 (Thermofisher Scientific, Massachusetts, USA), and anti‐rabbit Alexa Fluor 594 (Thermofisher Scientific, Massachusetts, USA).

The Masson's trichrome staining was performed using a Masson's trichrome stain kit (Abcam, Berlin, Germany) as per protocol.

H&E staining were performed using Eosin G solution 0.5% in water (Carl Roth GmbH, Karlsruhe, Germany) to stain the ECM and Hematoxylin solution (Carl Roth GmbH, Karlsruhe, Germany) to counterstain the nuclei. Images were acquired using Vectra 3.0 Microscope Automated Quantitative Pathology Imaging.

For each stained TEVGs’ section, representative images were acquired using an Axio Imager M2 fluorescence microscope equipped with an AxioCam MRm Rev.3 camera (Carl‐Zeiss, Oberkochen) with a magnification of 20×.

### Statistical Analysis

All in vitro samples were analyzed in triplicate (sample size = 3). To obtain R2 and R1 relaxation rate values, MRI images were processed with Imalytics Preclinical Software (Gremse‐IT GmbH, Aachen, Germany), which was used first to draw the ROI and then to obtain the R2 and R1 values, respectively. Colormaps were also obtained by using Imalytics Preclinical Software (Gremse‐IT GmbH, Aachen, Germany). Regarding the US analysis of bound MBs, images acquired in NLC mode were processed by drawing ROI using the VevoLAB software version 3.2 (FUJIFILM VisualSonics, Toronto, Ontario, Canada). After obtaining Contrast mean Power values for the corresponding drawn ROI, d.T.E values were obtained by subtracting the signal before and after MB destruction. The results are presented as mean values ± standard deviations (SD). The analysis was performed by using GraphPad Prism 9 (GraphPad Prism Software, San Diego, California, USA), and the statistical analyses between two groups (*t*‐test) or multiples groups (one‐way ANOVA) (one‐tailed) with the suggested post‐hoc correction test (Tukey) were applied as recommended by the software. Statistical significance was considered for values p ≤ 0.05.

## Conflict of Interest

The authors declare no conflict of interest.

## Supporting information

Supporting Information

## Data Availability

The data that support the findings of this study are available in the supplementary material of this article.
