# Supplementary material for: Monitoring the Remodeling of Biohybrid Tissue‐Engineered Vascular Grafts by Multimodal Molecular Imaging
Source: Adv Sci (Weinh). 2022 Feb 4;9(10):2105783. doi: 10.1002/advs.202105783 (PMC8981893; doi:10.1002/advs.202105783)
Supplement: Supplementary file 1 — Supporting Information [file ADVS-9-2105783-s001.pdf]

ADVANCED SCIENCE
Open Access

Supporting Information

for *Adv. Sci.,* DOI: 10.1002/advs.202105783

Monitoring the Remodeling of Biohybrid Tissue-Engineered Vascular Grafts by Multimodal Molecular Imaging

*Elena Rama, Saurav Ranjan Mohapatra, Christoph Melcher, Teresa Nolte, Seyed Mohammadali Dadfar, Ramona Brueck, Vertika Pathak, Anne Rix, Thomas Gries, Volkmar Schulz, Twan Lammers, Christian Apel, Stefan Jockenhoevel and Fabian Kiessling**

Supporting Information

Monitoring the Remodeling of Biohybrid Tissue-Engineered Vascular Grafts by Multimodal Molecular Imaging

Elena Rama, Saurav Ranjan Mohapatra, Christoph Melcher, Teresa Nolte, Seyed Mohammadali Dadfar, Ramona Brueck, Vertika Pathak, Anne Rix, Thomas Gries, Volkmar Schulz, Twan Lammers, Christian Apel, Stefan Jockenhoevel and Fabian Kiessling[*]

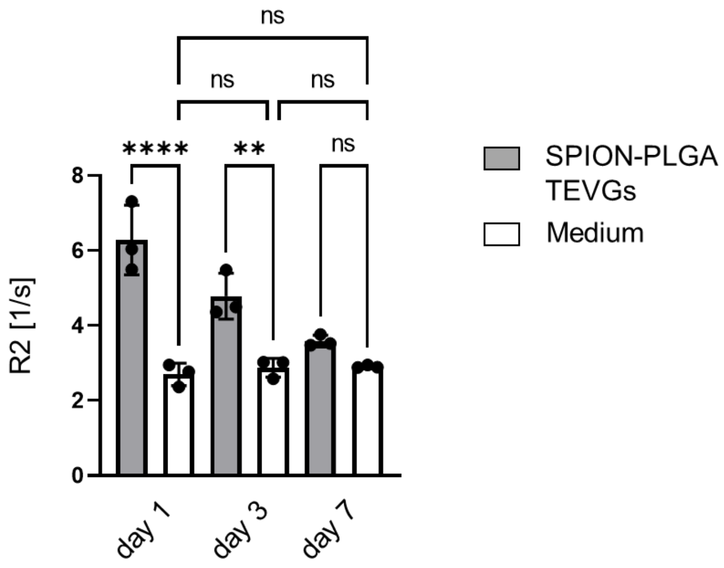

Figure S1. MRI evaluation of SPION release into the bioreactor medium. A slight increase in the R2 relaxation rates of the mediums was observed from day 1 to day 7 days of bioreactor conditioning of TEVGs, indicating the release of the SPION from the degrading PLGA fibers into the medium. Inversely, the R2 values of 0.2% SPION-labeled PLGA fibers decrease over time, indicating PLGA degradation. All values are obtained in triplicates: mean ± SD; *p<0.05, **p<0.01, ***p<0.001, and ****p<0.0001.

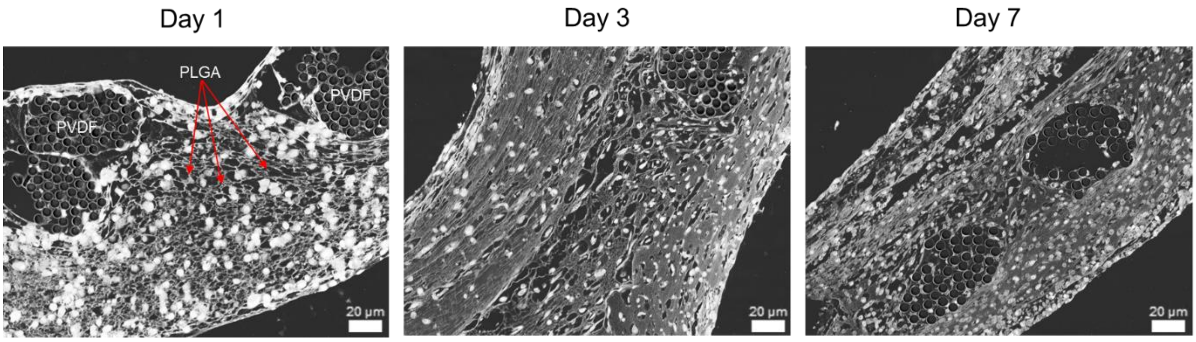

Figure S2. H&E staining of TEVGs. H&E staining were performed using Eosin G solution 0.5 % in water to stain the ECM and Hematoxylin solution for the counterstain of the nuclei. Images were acquired using Vectra® 3.0 Microscope Automated Quantitative Pathology Imaging System and converted in gray scale to enhance the visualization of the PLGA fiber. Histology images show the gradual replacing of PLGA with newly deposited ECM. Images were acquired after 1 day, 3 days, and 7 days of bioreactor conditioning of TEVGs.

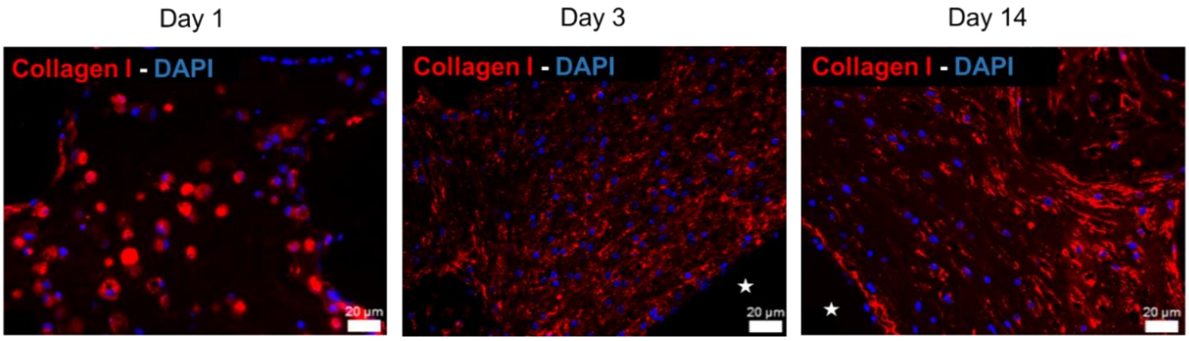

Figure S3. Histological analysis of collagen type I deposition within TEVGs. Immunofluorescence images show collagen type I positive cells already at day 1 of TEVGs maturation. At day 3 of bioreactor conditioning, there is a strong collagen type I deposition, which further increases until the last day of TEVGs monitoring. The star indicates TEVGs' lumen.

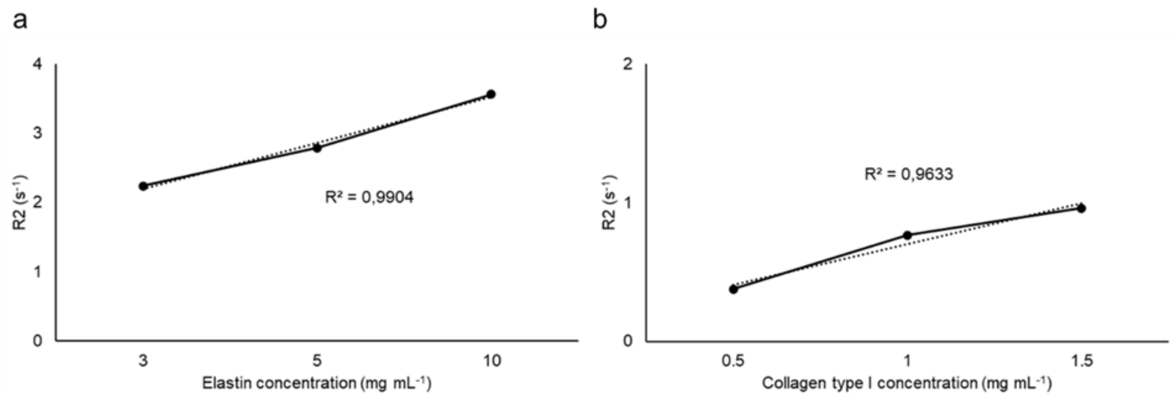

Figure S4. Linearity between MRI signal and amount of elastin and collagen type I. Graphs show the linear correlation between the ESMA or EP-3533 MRI signal at different concentration of elastin or collagen type I introduced in the fibrin gel used for the molding of the vascular grafts.

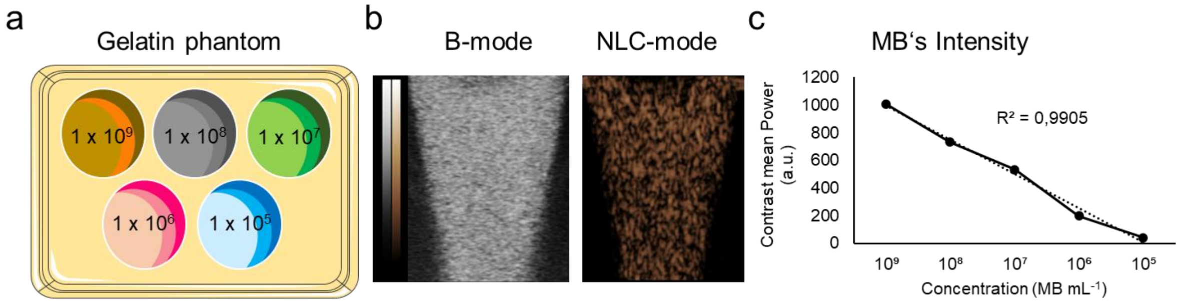

Figure S5. Linear correlation between MB's concentration and US contrast intensity. a: Schematic depiction of the 10% w/v gelatin phantom in which the wells were obtained by dipping sand-filled Eppendorf tubes until solidification of the gelatin. Later, the wells were filled with a mixture of 3% w/v gelatin and different concentration (ranging from 1 x 10^9 MB mL^{-1} to 1 x 10^5 MB mL^{-1}) of c[RGDfK]-MBs. b: US images in B-mode and NLC-mode of the gelatin embedded MBs. c: The linear correlation between MB's concentration and US signal intensity detected (R^2 = 0.9905) indicates the high sensitivity of the US.

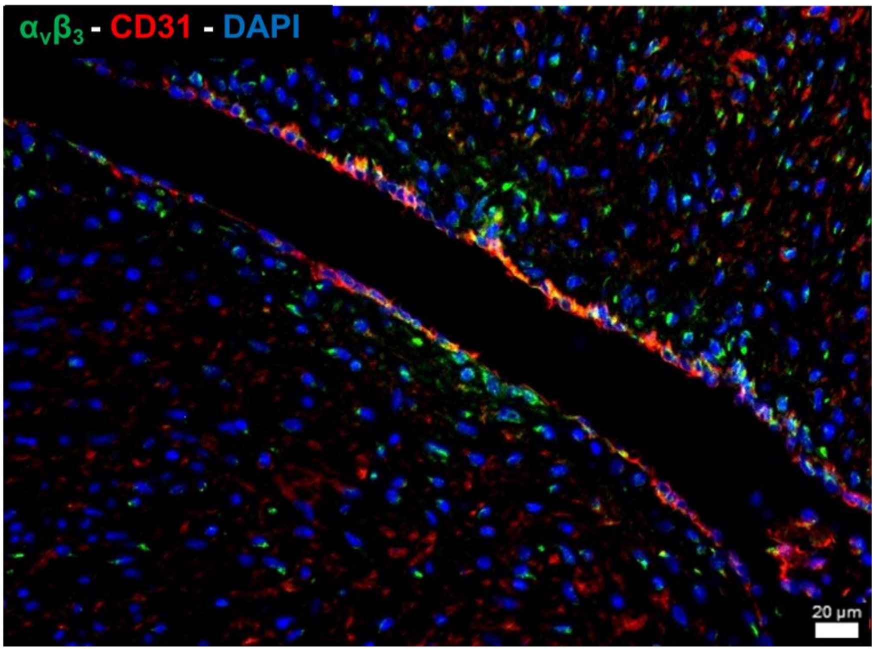

Figure S6. $\alpha_v\beta_3$ integrin staining (green) in HUA. ECs are visualized with a CD31 staining (red) and cell nuclei with DAPI (blue). This representative image shows the colocalization of $\alpha_v\beta_3$ integrins with the endothelial lining, indicating the physiological expression of integrins within the HUA.
